# IL-17 Cytokines and Chronic Lung Diseases

**DOI:** 10.3390/cells11142132

**Published:** 2022-07-06

**Authors:** Felix Ritzmann, Lars Peter Lunding, Robert Bals, Michael Wegmann, Christoph Beisswenger

**Affiliations:** 1 Department of Internal Medicine V—Pulmonology, Allergology and Respiratory Critical Care Medicine, Saarland University, 66421 Homburg, Germany; felix.ritzmann@uks.eu (F.R.); robert.bals@uks.eu (R.B.); 2 Helmholtz Institute for Pharmaceutical Research, 66123 Saarbrücken, Germany; 3 Division of Lung Immunology, Priority Area Asthma and Allergy, Research Center Borstel—Leibniz Lung Center, 23845 Borstel, Germany; llunding@fz-borstel.de (L.P.L.); mwegmann@fz-borstel.de (M.W.); 4 Airway Research Center North (ARCN), German Center for Lung Research (DZL), 23845 Borstel, Germany

**Keywords:** IL-17, lung diseases, inflammation, lung damage, infection

## Abstract

IL-17 cytokines are expressed by numerous cells (e.g., gamma delta (γδ) T, innate lymphoid (ILC), Th17, epithelial cells). They contribute to the elimination of bacteria through the induction of cytokines and chemokines which mediate the recruitment of inflammatory cells to the site of infection. However, IL-17-driven inflammation also likely promotes the progression of chronic lung diseases, such as chronic obstructive pulmonary disease (COPD), lung cancer, cystic fibrosis, and asthma. In this review, we highlight the role of IL-17 cytokines in chronic lung diseases.

## 1. Introduction

**Figure 1:**
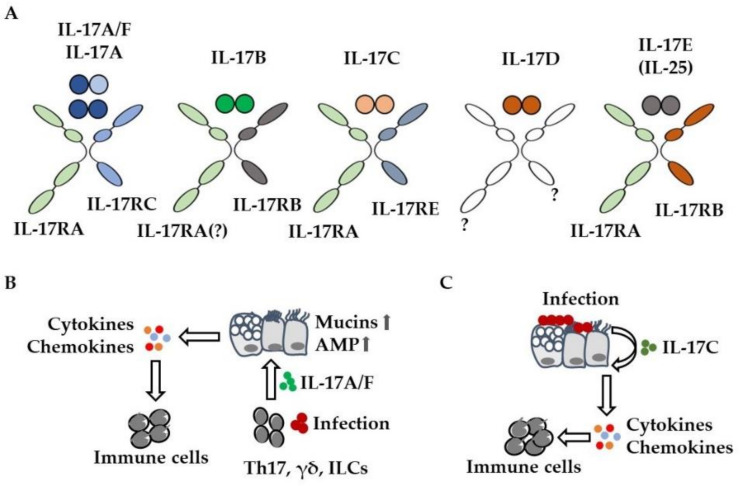
IL-17 cytokines mediate the recruitment of immune cells. (**A**) IL-17 cytokine and receptor family. To which receptors (?) IL-17B and IL-17D bind remains to be elucidated. (**B**) Infection results in the release of IL-17A from immune cells (γδ T, innate lymphoid (ILC), Th17 cells). IL-17A activates lung epithelial cells, resulting in enhanced chemokine-mediated recruitment of immune cells (e.g., neutrophils) and enhanced expression (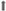) of mucins and antimicrobial peptides (AMP). (**C**) Bacteria and viruses induce the expression of IL-17C in lung epithelial cells. IL-17C enhances the expression of cytokines and chemokines in an autocrine manner, resulting in increased pulmonary inflammation.

Interleukin 17 (IL-17) cytokines are key inflammatory mediators in numerous diseases such as autoimmunity, allergy, and infection [1]. The first and best characterized IL-17 cytokine is IL-17A, which was originally described as being released by T helper 17 (Th17) cells during an adaptive immune response. Subsequently, five additional members (IL-17B, -C, -D, -E, and -F) could be identified (Figure 1A). The amino acid sequence identity of the six IL-17 cytokines ranges from 16 to 50%. All IL-17 cytokines harbor five conserved cysteine residues at the C-terminus and assemble into homodimers [2]. In addition, IL-17A can form a heterodimer together with IL-17F. By binding to corresponding receptors (IL-17RA to IL-17RE), IL-17 cytokines activate signaling cascades that mediate the expression of inflammatory mediators (e.g., antimicrobial peptides, cytokines, and chemokines) in target cells. In this way, IL-17 cytokines indirectly orchestrate the recruitment of inflammatory cells to the site of inflammation [3].

IL-17A signals through a receptor complex of IL-17RA/IL-17RC, whereas IL-17C acts as a homodimer through IL-17RA/IL-17RE [4] (Figure 1A). As both cytokines require IL-17RA, there is a functional overlap between IL-17A and IL-17C. IL-17A and IL-17C promote the expression of cytokines, chemokines, and antimicrobial peptides in target cells (e.g., lung epithelial cells) through the transcription factor NF-κB (nuclear factor kappa-light-chain-enhancer of activated B cells), MAP (mitogen-activated protein) kinases, and the stabilization of mRNA and act in synergy with additional inflammatory mediators (e.g., tumor necrosis factor (TNF)-α) or microbial stimuli [5]. IL-17A and IL-17F are expressed by immune cells including gamma delta (γδ) T, innate lymphoid, and Th17 cells, whereas IL-17C is primarily expressed by epithelial cells (Figure 1B,C) [3,5]. A variety of preclinical studies showed that IL-17A contributes to the elimination of lung pathogens (e.g., *Streptococcus pneumoniae*, *Klebsiella pneumoniae*) through the recruitment of inflammatory cells. However, IL-17A-mediated recruitment of inflammatory cells such as neutrophils likely contributes to lung damage in severe pneumonia and acute respiratory distress syndrome (ARDS) [3,6,7,8,9,10]. IL-17A also induces mucus production and goblet cell metaplasia in lung epithelial cells, a common hallmark for asthma and cystic fibrosis (CF) [3,11,12,13] (Figure 1B).

IL-17C promotes inflammation in an autocrine manner but can also signal to T helper cells as well [14,15,16,17]. Preclinical studies showed that bacteria and viruses induce the expression of IL-17C in lung epithelial cells within hours and that IL-17C contributes neutrophil chemotaxis and acute neutrophilic inflammation in lungs infected with *S. pneumoniae* and *P. aeruginosa* (Figure 1C) [15,16,18,19,20,21,22,23]. While a multitude of studies have addressed the role of IL-17A, -C, and -F in lung diseases, the remaining IL-17-cytokines have received little attention so far. IL-17E (also known as IL-25) which binds to IL-17RA/IL-17RB may have a role in the development of allergic lung disease. A recent study, for instance, suggests that IL-17E derived from tuft cells—a rare epithelial cell type—promotes type 2 inflammation in the airways [24]. The role of IL-17B and IL-17D in acute and chronic lung disease has not been studied so far.

Loss of lung structure and function are common hallmarks of diverse chronic lung diseases (e.g., chronic obstructive pulmonary disease (COPD), cystic fibrosis (CF) and asthma). Airway remodeling including mucus hyperproduction, goblet cell metaplasia, bronchial wall thickening, airway smooth muscle cell proliferation, and increased extracellular matrix deposition can be observed in these diseases [25]. This leads to narrowing of the airways and shortness of breath. In addition, the destruction of the lung parenchyma, especially in COPD, can lead to pulmonary emphysema, which is accompanied by a massive loss of the gas exchange surface [26]. Inflammation, often caused by acute and persistent lung infections, is a major driver of these symptoms [27]. Inflammation is also an important cause of cancer development and progression [28]. Although IL-17 cytokines mediate the elimination of pathogens in the lung, they are suspected to contribute to the progression of lung disease by promoting and exacerbating excessive inflammation. In the following sections, we highlight the role of IL-17 cytokines in the development and progression of chronic lung diseases.

## 2. Asthma

Asthma is a chronic inflammatory disease of the airways that actually affects more than 260 million people worldwide [29]. Its symptoms comprise productive cough, wheezing, shortness of breath, chest tightness, and a variable degree of broncho-obstruction, which is typically associated with airway hyperresponsiveness [30]. Among different patients, the severity, duration, and frequency of these symptoms show a considerable degree of variability, which is also true for the response to corticoid therapy. Thus, asthma can be clinically determined by its severity of clinical symptoms (mild, moderate, and severe), by its responsiveness to corticoid therapy (corticoid responsive or resistant, controlled or uncontrolled, respectively), by its type of inflammation (eosinophilic, neutrophilic, and paucigranulocytic), and by the evidence of cytokines that can be attributed to T helper 2 type immune responses (T2) or not (non-T2) [31,32,33]. This pronounced heterogeneity led to the formulation of several phenotypes and endotypes of asthma, which is actually understood as a syndrome rather than a distinct disease.

Most cases of asthma reveal elevated numbers of eosinophils in sputum and peripheral blood samples and display increased levels of T helper 2 type cytokines such as IL-4, IL-5, and IL-13. Usually, patients with this eosinophilic T2 asthma respond to corticoid therapy and display mild-to-moderate symptoms [34]. In contrast, non-T2 asthma is often associated with neutrophilic or a combination of neutrophilic and paucigranulocytic inflammatory categories, and is more common among those patients that display corticoid resistance and comorbidities such as obesity [35]. Consequently, non-T2 asthma tends to present more severe symptoms, lower controllability of symptoms, and a higher risk for exacerbations [36].

### 2.1. IL-17A and IL-17F in Asthma

The expression and production of IL-17A and IL-17F is enhanced in patients with asthma. Thus, increased levels of these cytokines have been observed in sputum, serum, bronchial, and nasal biopsies [37,38,39,40,41,42,43]. Thereby, IL-17A sputum levels are negatively correlated with airway responsiveness to methacholine [44], while IL-17A expression levels in bronchial biopsies correlate with the degree of airway neutrophilia [36,41,45] and are more pronounced in patients with moderate-to-severe or exacerbation-prone asthma than in those with mild-to-moderate asthma [36,40,42,46,47]. IL-17F levels are higher in both mild-to-moderate and severe asthma patients as compared to healthy controls [42]. Since the increased expression of IL-17A in asthma patients has been attributed to T cells that were later described as Th17 cells [41], this non-T2 asthma endotype has also been termed Th17-high asthma. On the one hand, Th17-high asthma displays a considerable low therapeutic response to both corticoid and biologicals directed against Th2 type cytokines, and on the other hand, it is commonly referred to as neutrophilic asthma [36,40,41,46,48,49]. Consequently, therapy or symptom control of this asthma endotype still represents an unmet medical need and led to detailed research efforts to untangle the relationship between IL-17, Th17 cells, neutrophils, and corticosteroid resistance in asthma.

Since neutrophils do not express IL-17RC, they cannot directly respond to IL-17A or IL-17F [50], which suggests an indirect effect of IL-17A on airway neutrophilia. Indeed, IL-17A appears to mainly act on structural cells of the airway mucosa. Thus, in vitro stimulation of bronchial epithelial cells, endothelial cells, fibroblasts, and airway smooth muscle cells results in the production and release of the potent neutrophil attractors CXCL1 (growth-related oncogene (GRO) α), CXCL5 (epithelial cell-derived neutrophil-activating protein (ENA) 78), and CXCL8 (IL-8), as well as of growth factors including granulocyte-macrophage colony stimulating factor (GM-CSF) and granulocyte colony-stimulating factor (G-CSF), and of proinflammatory cytokines such as IL-1, IL-6, IL-11, and TNF, which creates an ideal microenvironment for neutrophil infiltration [37,39,50,51,52]. Kawaguchi et al., demonstrated in detail that IL-17F provides comparable effects, since it stimulates the release of IL-1, IL-6, CXCL1, CXCL5, and CXCL8 in bronchial epithelial cells as well as in endothelial cells [39,53,54,55,56]. In line with this, instillation of either IL-17A or a vector inducing IL-17F overexpression results in prominent airway neutrophilia in mice [57,58], which can be specifically inhibited by application of, e.g., an antibody against IL-17A [59].

The study of McKinley et al., was the first to demonstrate that IL-17-producing Th17 cells are able to promote airway neutrophilia and corticoid resistance by using adoptive transfer of in vitro-differentiated Th17 cells from DO11.10 TCR-transgenic mice to mice undergoing induction of ovalbumin (OVA)-induced experimental allergic asthma [60]. This mouse model typically reflects eosinophilic mild-to-moderate T2 asthma and does not display a significant increased release of IL-17A or IL-17F. In contrast, other asthma models mimicking more neutrophilic or severe asthma endotypes clearly demonstrated a pathological role for the IL-17A/IL-17F/IL-17RC axis in the modeled diseases. Hence, mice with experimental asthma induced by local application of house dust mite extract (HDM) display not only prominent airway neutrophilia but also increased release of IL-17A and infiltration of Th17 cells into the airways. In this model, inhibition of Th17 cell differentiation and, thus, reduction of IL-17A release significantly lowered infiltration of neutrophils into the airways and pathological hallmarks of HDM-induced experimental asthma [61,62]. Chenu et al., also reported a significant role for IL-17F in this setting [63]. Mice with *Aspergillus fulmigatus*-induced allergic airway inflammation also display infiltration of Th17 cells and production of IL-17 cytokines [64]. De Luca et al., used mice deficient in either IL-17A, IL-17F, or IL-17RA in this model to unriddle for contribution of these cytokines to the pathogenesis of the disease and clearly demonstrated the contribution of the IL-17F/IL-17RC-axis to the development of allergic airway inflammation [65]. Furthermore, in a mouse model of steroid-insensitive toluene diisocyanate-induced asthma, it appears that IL-17F is the driving force behind neutrophil infiltration into the airways and resistance to corticosteroids [66].

However, the pathological role of IL-17 cytokines seems not to be reduced solely to the induction of corticoid-resistant airway neutrophilia. IL-17A is also able to induce the expression of the mucin genes *MUC5AC* and *MUC5B* in bronchial epithelial cells in vitro [67], and enhanced expression of IL-17A correlates with MUC5AC expression in a mouse model experimental asthma after infection with respiratory syncytial virus (RSV) [68]. Comparable effects could be observed for IL-17F, when this cytokine is overexpressed in mice with experimental allergic asthma [58]. Additionally, IL-17A and IL-17F could be implemented in the development of airway remodeling. Thus, IL-17A triggers the release of IL-6 and IL-11, which exert profibrotic activities, and IL-17F has been shown to induce the expression of the potent profibrotic factor transforming growth factor (TGF) [37,69].

Remarkably, although Th17 responses have been reported to counter-regulate Th2 responses and vice versa [61,70,71,72], under certain conditions IL-13 and IL-17A appear to act synergistically on the inflammatory response underlying asthma formation. Hence, IL-17A has been shown in vitro to enhance IL-13 activity by enhancing IL-13-induced signal transducer and activator of transcription 6 (STAT6) activation and augmented airway hyperresponsiveness, mucus production, airway inflammation, and IL-13-induced gene expression in vivo compared with mice given intratracheal IL-13 alone [73]. In line with that, simultaneous induction of IL-13 and IL-17A led to pronounced airway hyperresponsiveness (AHR) in a mouse model of experimental asthma [61]. Combined neutralization of both IL-13 and IL-17A diminished the pathological signs of Th2/Th17 high experimental asthma in mice [74]. Furthermore, local application of polyinosinic:polycytidylic acid (polyIC) to mice with OVA-induced experimental allergic asthma led to infiltration of IL-17A, producing natural killer (NK) cells and expression of IL-17C, which mediate exacerbation of airway inflammation, mucus hyperproduction, and AHR [75,76]. Since lipopolysaccharide (LPS)-induced exacerbation of experimental asthma in mice is also associated with increased release of IL-17A and can be blocked by an antibody directed against this cytokine, IL-17A could indeed play a significant role during acute asthma exacerbations triggered by bacteria as well as respiratory viruses [77].

These multiple implications in the pathogenesis of asthma and the promising findings in preclinical mouse models indicated that neutralization of IL-17A effects could be a novel option to treat severe, neutrophilic, or uncontrolled asthma. Thus, Busse et al., evaluated the efficacy and safety of brodalumab, a human anti-IL-17 receptor A monoclonal antibody, in patients with inadequately controlled moderate-to-severe asthma. At a first glance, the outcome of this study was rather disappointing. Apart from an ACQ (asthma control questionary) change in the high-reversibility subgroup only, the authors did not observe any treatment differences for the overall study population [78]. A closer look at the study design reveals that the authors took the heterogeneity of the severe asthma population into account, but did not include production levels of IL-17A, that is, the cytokine that should be neutralized by the tested biological. Why this assessment was omitted remains elusive and leaves the possibility that an anti-IL-17A-directed therapeutic approach still could provide beneficial effects in those severe asthma patients that display an IL-17/Th17 high endotype. Consequently, further experimental approaches to target IL-17A are under investigation. Cyanidin, a natural small molecule, has been demonstrated to block IL-17RA and, thus, to inhibit IL-17A binding to its receptor and revealed therapeutic effects in a mouse model of experimental asthma [79]. Two other small molecules, namely CBG040591 and CBG060392, have also been described as interfering with IL-17A binding to IL-17RA and diminishing the IL-17A-induced production of CXCL8 and IL-6 in vitro [80]. Though such approaches have therapeutic potential, they still have the disadvantage of targeting only one of the multiple cytokines that orchestrate the inflammatory response underlying the formation of (severe) asthma. Consequently, approaches that target more of these cytokines or more upstream events in the inflammatory cascades theoretically provide stronger therapeutic effects as has been shown, e.g., for targeting of the transcription factor GATA-3 in T2 asthma [81,82]. Thus, retinoic acid receptor-related orphan nuclear receptor gamma (RORγt), the fate-determining transcription factor of Th17 cells, has already been addressed by small molecules that inhibit binding to its target loci and, thus, prevent Th17 cell differentiation and release of proinflammatory cytokines such as IL-17A and IL-22 [83,84].

### 2.2. IL-17E (IL-25) in Asthma

An increased expression of IL-17E (IL-25) has been observed in the bronchial mucosa and patients with atopic asthma after allergen challenge [85] and its levels in sputum correlate with disease severity [86]. Its receptor, constituted by IL-17RB and IL-17RA, is expressed by various cells contributing to asthma pathogenesis, such as T cells, ILCs, and inducible natural killer (iNKT) cells, as well as by structural cells including epithelial cells, fibroblasts, and endothelial cells [87]. While overexpression of IL-17E in the lung resulted in significantly enhanced antigen-induced Th2 cell infiltration and T2 type cytokine production, pronounced airway eosinophilia, goblet cell hyperplasia in the airways, and airway remodeling, neutralization of this cytokine either by application of a soluble IL-17E receptor or by an anti-IL-17E-antibody lowered allergic airway inflammation and pathological features of experimental asthma in mice [88,89]. Gregory et al., found that the secretion of innate epithelial-derived cytokines IL-33 and thymic stromal lymphopoietin (TSLP) was completely ablated after neutralization, which indicates that IL-17E drives the production of these cytokines that act as potent facilitators of Th2 cell differentiation. Furthermore, IL-17E has been shown to stimulate the release of Th2-related cytokines, such as IL-4, IL-5, and IL-13 in IL-17RB expressing inducible NKT cells [90], and the secretion of IL-4 in group-2 innate lymphoid tissue cells (ILC2) [91,92], and to augment Th2 cell differentiation in an IL-4-dependent fashion [93]. Taken together, these findings indicate that IL-17E acts differently to its relatives IL-17A and IL-17F in asthma pathogenesis. By supporting the release of IL-33 and TSLP and the production of Th2-related cytokines, it efficiently promotes Th2 cell development and thus favors allergic inflammation of the airways. Thus, it has been described as an alarmin that is mainly implicated in the development of T2 asthma; however, clinical studies investigating the putative therapeutic effect of anti-IL-17E in asthma patients have not been published yet.

## 3. COPD

Cigarette smoke (CS) and indoor air pollution in developing countries are the major risk factors for COPD. COPD is characterized by chronic pulmonary inflammation, airflow limitation, progressive loss of lung function, and emphysema [94]. In the course of the disease, bacteria (e.g., nontypeable *Haemophilus influenzae* (NTHi)) often chronically infect lungs of COPD patients. Moreover, bacterial and viral infections trigger acute exacerbations of COPD (AECOPD) which are characterized by sudden worsening of airway function and overall symptoms [95]. Thus, it is suggested that acute and chronic infections drive pulmonary inflammation in a vicious cycle of impaired lung defense, infection, inflammation, and loss of lung function and structure [95].

### 3.1. IL-17A in Stable COPD

As IL-17 cytokines mediate the recruitment of inflammatory cells, such as neutrophils, a variety of preclinical studies examined a possible role for IL-17A and -F and its receptors in COPD. Roos et al., showed that numbers of IL-17A-, IL-17F-, IL-17RA-, and IL-17RC-expressing cells are increased in lung samples obtained from stable COPD patients with mast and T cells being the primary source of IL-17A in end-stage COPD and that the expression of IL-17A correlates with disease progression [96,97]. Increased numbers of IL-17A-expressing cells could also be detected in the bronchial mucosa and in the epithelium of the small airways of stable COPD patients compared with healthy control subjects [42,98]. Studies also suggest that concentration of IL-17A in blood obtained from COPD patients negatively correlates with lung function parameters, such as FEV1 (forced expiratory volume in 1 s) predicted [99,100,101]. However, comprehensive studies showing that IL-17A can serve as a blood biomarker for COPD are missing.

The role of IL-17A has further been examined in CS-, elastase-, and ozone-dependent experimental models of lung damage. Chen et al., showed that pulmonary inflammation and lung damage (determined by measuring the mean linear intercept) are reduced in IL-17RA-deficient mice exposed to CS for 6 months [102]. In line with this study, IL-17A deficiency protected mice from CS-induced decreases in lung density and increases in lung volume determined through quantitative microcomputed tomography after 16 weeks of CS exposure [103]. Similar results were obtained in models of elastase-induced lung damage. Elastase-induced inflammation and loss of lung structure were reduced in IL-17A-deficient mice as well as in mice treated with therapeutic anti-IL-17A antibodies [104,105,106]. Moreover, IL-17A deficiency resulted in a decreased apoptosis of alveolar type-II cells in mice exposed to CS for 4 weeks [107] and an attenuated lymphoid neogenesis in mice exposed to CS for 24 weeks [96]. However, contrary to the mentioned studies, in a study by Voss et al., IL-17A-deficient mice were not protected from CS-induced loss of lung structure and function determined after 6 months of CS exposure. Deficiency for IL-17RA also did not protect mice from an ozone-induced loss of lung structure [108]. Taken together, the function of IL-17A in the development of lung damage is dependent on the model used. As there are no standardized models, differences in the protocol used, such as duration of smoke exposure and composition of the smoke, may result in opposing outcomes.

Even though preclinical studies suggest that IL-17A drives disease progression, there is, so far, no strong evidence that IL-17A is a suitable target in COPD. In a randomized, placebo-controlled phase-2 trial performed by Eich et al., an anti-IL-17A monoclonal antibody given over a period of 12 weeks did not show significant effects in patients with moderate-to-severe symptomatic COPD. Therefore, the authors concluded that IL-17A is not an adequate therapeutic target in COPD [109]. However, it remains an open question whether targeting IL-17A is beneficial for specific COPD phenotypes, such as patients with an airway epithelial IL-17A response signature [45].

### 3.2. IL-17A during AECOPD

There is evidence that IL-17A promotes inflammation during AECOPD. Roos et al., analyzed concentrations of IL-17A and IL-17F in sputum obtained from COPD patients before (stable), during, and after resolution of AECOPD [9]. They found that concentrations of IL-17A, but not IL-17F, are increased in sputum during NTHi-associated acute exacerbations compared with samples before and after the exacerbation. Significantly increased concentrations of IL-17A were not determined in sputum samples in which NTHi was not detected [9]. A role for IL-17A in NTHi-triggered inflammation is further suggested by a study showing that ex vivo stimulation of lung tissue obtained from COPD patients with NTHi results in increased numbers of IL-17A producing T cells compared to non-COPD controls [110]. Moreover, neutrophilic lung inflammation was reduced in IL-17A-deficient mice as well as in anti-IL-17A antibody-treated mice exposed to CS for 4 days followed by infection with NTHi [9]. Antibody-mediated depletion of IL-17A also decreased RSV-mediated lung injury in mice treated before with a combination of elastase and LPS [111]. There is some evidence that IL-17A is increased in the blood of COPD patients during AECOPD [99]. However, carefully elaborated studies examining IL-17A as a reliable biomarker for AECOPD are missing.

### 3.3. IL-17C and COPD

Little is known about the remaining IL-17 cytokines in the context of COPD. There is some evidence for an increased expression of IL-17C in COPD. Immunohistochemistry revealed IL-17C expression in the bronchial mucosa of COPD patients [15]. Rao et al., identified a pathogenic stem cell strongly expressing IL-17C in lung tissue obtained from COPD patients [112]. Vella et al., showed that concentrations of IL-17C in sputum samples obtained during AECOPD are associated with disease severity [113]. However, sputum from only 36 patients was analyzed and stable patients were not included in the study.

Moreover, recent studies showed that lung pathogens that cause AECOPD and chronically infect lungs of stable COPD patients (e.g., NTHi, *P. aeruginosa*, rhinovirus) induce the expression of IL-17C in lung epithelial cells. Importantly, cultured bronchial epithelial cells obtained from COPD patients produced increased amounts of IL-17C after stimulation with a combination of rhinovirus and NTHi compared to nonsmokers and healthy smokers [21]. IL-17C further increases the expression of cytokines and chemokines induced by AECOPD-associated pathogens in an autocrine manner [14,15,16,18,20,21,22,23,114]. Knockdown of IL-17C in bronchial epithelial cells with small interfering RNAs (siRNAs) resulted, for instance, in a significantly decreased expression of chemokines as well as neutrophil chemotaxis induced by the stimulation with the combination of rhinovirus and NTHi [21]. However, tissue culture experiments also showed that CS suppresses the pathogen-induced expression of IL-17C in respiratory epithelial cells [15,21]. Thus, suppressed expression of IL-17C may promote infections in smokers.

The function of IL-17C was also examined in CS- and NTHi-dependent experimental models of lung damage. In line with the mentioned cell culture studies, chemokine expression and neutrophilic lung inflammation were decreased in IL-17C-deficient mice chronically exposed to NTHi, whereas deficiency for IL-17C did not affect CS-induced lung inflammation [113]. Moreover, NTH-induced loss of lung parenchyma was significantly decreased in IL-17C-deficient mice [113]. Further studies are needed to clarify whether viruses and bacteria induce the expression of IL-17C during AECOPD and whether IL-17C is a suitable therapeutic target in COPD, especially during AECOPD.

## 4. Lung Cancer

Chronic inflammation as seen in COPD patients contributes to the initiation and progression of lung cancer [115]. Lung tumors are infiltrated with a variety of immune cells including neutrophils and macrophages [116]. There is evidence that levels of IL-17A and IL-17A-producing cells in the tumor microenvironment and bloodstream are associated with disease progression in lung cancer patients [117,118,119,120]. Therefore, in recent years, IL-17A has been studied in a variety of preclinical lung cancer models.

Murine models of KRAS-driven lung cancer suggest that IL-17A and IL-17A-expressing lymphocytes mediate tumor-associated inflammation and tumor cell proliferation. Chang et al., showed that IL-17A deficiency, but not IL-17F deficiency, results in reduced tumor growth and tumor-associated inflammation in the presence and absence of NTHi-induced lung inflammation [121]. Studies further showed that the forced expression of IL-17A in the lung promotes tumor proliferation through IL-6 and tumor-associated neutrophils and that IL-17A mediates resistance to therapeutic programmed cell death protein 1 (PD-1) blockade [120,122]. Jin et al., studied the tumor-promoting effect of the commensal microbiota in a model in which the tumor suppressor tumor protein p53 is deleted in lung epithelial cells in addition to the activation of oncogenic Kras. In this model, the commensal microbiota drives tumor-associated inflammation (e.g., the recruitment of neutrophils into the tumor microenvironment) and tumor progression through IL-17A expressed by γδ T cells [123]. However, in a model in which the deletion of phosphatase and tensin homolog deleted on chromosome ten (Pten) and SMAD family member 4 (Smad4) from airway epithelial cells results in the development of spontaneous tumors, tumor incidence was increased in mice deficient for IL-17A [124]. Thus, the role of IL-17A is possibly dependent on the nature of the tumor.

There is also evidence that IL-17A affects the formation of lung metastases. Coffelt et al., showed in murine models that systemic inflammation induced by mammary tumors promotes lung metastases through IL-17A expressing γδ T cells and neutrophils [125]. Moreover, treatment with IL-17A neutralizing antibodies resulted in a decreased lung tumor growth in mice intravenously injected with Lewis lung carcinoma cells [126]. However, in other models, IL-17A counteracted the growth of metastases. The formation of lung metastasis was increased in mice deficient in IL-17A intravenously inoculated with the colon cancer cell line MC38 [127]. Moreover, Martin-Orozco et al., showed that tumor growth is increased in IL-17A-deficient mice injected intravenously with B16-F10 melanoma cells and that Th17 cells mediate the activation of tumor-specific cytotoxic T cells [128].

There are only a few studies concerning the role of the remaining IL-17 cytokines in lung cancer. Yang et al., found that IL-17RB expression in tumor tissue measured via immunostaining associates with lymph node metastasis, distant metastasis, and poor patient survival in a cohort of 139 lung cancer patients. Overexpressing IL-17RB in cancer cell lines resulted in an increased migration in tissue culture experiments and an increased tumor burden in mice after intravenous injection of the cells [129]. Jungnickel et al., identified the expression of IL-17C in tumor tissue as a negative prognostic factor for lymph node metastasis in a cohort of 103 non-small cell lung cancer patients. In the Lewis lung carcinoma cell model, IL-17C mediated NTHi-induced neutrophilic lung inflammation and lung tumor growth [130]. Ritzmann et al., demonstrated that IL-17C mediates tumor-associated inflammation and tumor growth in KRAS-driven lung cancer. IL-17C deficiency further resulted in an increased response to PD-1 blockade in mice with chronic NTHI-induced inflammation of the lung [131]. In a spontaneous breast tumor model, treatment with an IL-17E-neutralizing antibody resulted in reduced growth of lung metastasis without affecting the primary tumor. In this model, tumor-infiltrating macrophages and cluster of differentiation 4 (CD4) cells were identified as expressing IL-17E in the primary mammary adenocarcinoma [132].

In summary, additional studies are required to clarify whether and how the role of IL-17 cytokines in the progression of primary lung cancer and formation of metastases depends on the nature of the tumors. Targeting IL-17 cytokines or pathways in combination with additional therapeutic approaches, such as immune checkpoint inhibitors, could be an interesting option in defined lung cancer phenotypes [133]. A potential use for IL-17 as a biomarker in lung cancer needs further evaluation.

## 5. Cystic Fibrosis

Cystic fibrosis (CF) is caused by different mutations in the CF transmembrane conductance regulator (CFTR) gene. Viscous secretions in the airways, impaired mucociliary clearance, microbial infections (e.g., with *P. aeruginosa, Staphylococcus aureus*), and chronic neutrophilic lung inflammation result in loss of lung structure and function in CF [134]. A variety of studies have analyzed the expression of IL-17A and IL-17A-expressing cells in samples obtained from CF patients. Decraene et al., showed that IL-17A concentrations as well as *IL-17A* mRNA expression are increased in the induced sputum of stable CF patients compared with healthy control subjects [135]. IL-17A concentrations were also found to be increased in bronchoalveolar lavage (BAL) fluids of CF patients and to correlate positively with numbers of BAL neutrophils [136,137,138]. In addition, levels of cells positive for IL-17A have been described to be augmented in lung tissue of CF patients, with lymphocytes being likely the main source of IL-17A and IL-17F [136,139,140]. Hagner et al., showed that a variety of lymphocytes including Th17 cells, CD3^+^ CD8^+^ T-cells, innate lymphoid cells, γδ T cells, and NK cells secrete IL-17A into the lungs, lymph nodes, and blood of CF patients [140]. Moreover, Golebski found that IL-17A-secreting innate lymphoid 2 cells are present in nasal polyps of CF patients and contribute to neutrophilia through the induction of IL-8 in epithelial cells [141].

Acute and chronic lung infections with *P. aeruginosa* are key drivers of disease progression and mortality [134]. Thus, the role of IL-17A in *P. aeruginosa*-induced lung inflammation has been examined in preclinical studies. Numerous mouse studies showed that infection with mucoid and non-mucoid *P. aeruginosa* strains is associated with increased pulmonary expression of IL-17A [18,139,140,142,143,144,145]. In such models, IL-17A and IL-17RA deficiency results in decreased control of chronic lung infection [142,144]. However, preclinical studies also demonstrated that treatment of mice with an anti-IL-17A antibody before or after chronic infection had been established decreases neutrophilic inflammation of the lung [142,145]. Moreover, Hagner et al., studied the function of IL-17A in β-epithelial Na^+^ channel transgenic (Scnn1b-Tg) mice [140]. These mice express IL-17A in γδ T cells as well as innate and adaptive lymphocytes and exhibit CF-typical characteristics such as neutrophilic inflammation, lung damage, and airway mucus plugging. Ablation of IL-17A in Scnn1b-Tg mice resulted in reduced neutrophilic airway inflammation and structural lung damage, but did not affect mucus obstruction [140].

Analysis of samples obtained from patients together with mouse studies suggests that antibody-mediated blockade of IL-17A could be a therapeutic strategy to decrease lung inflammation in CF. However, IL-17A in combination with TNF-α also enhances the efficacy of CFTR modulators [146,147]. Further studies are needed to elucidate under which conditions therapeutic approaches addressing IL-17-signaling can be beneficial for CF patients.

## 6. Conclusions

**Figure 2:**
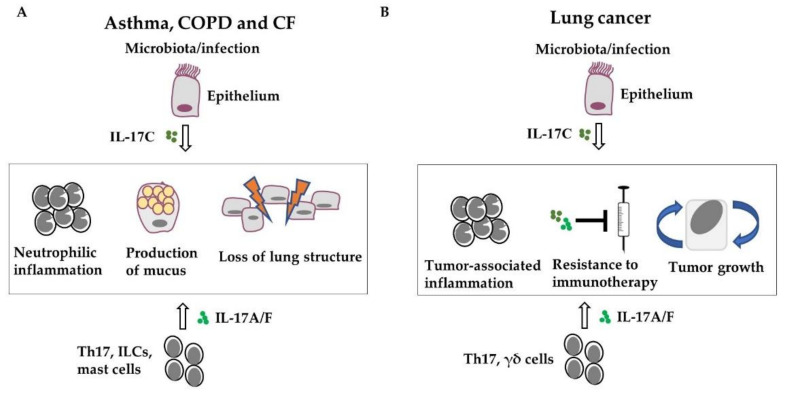
IL-17 cytokines promote the progression of chronic lung diseases. Acute and chronic infections or disease-associated microbiota drive the expression of IL-17 in immune and epithelial cells. IL-17-mediated expression of cytokines and recruitment of inflammatory cells, mainly neutrophils, result in airway remodeling (e.g., goblet cell hyperplasia) and lung damage in COPD, CF, and asthma (**A**) as well as in tumor proliferation and resistance to immunotherapy in lung cancer (**B**).

Acute and chronic infections as well as a disease-associated microbiota are likely the main drivers of IL-17 cytokine expression in immune and epithelial cells in chronic lung diseases (Figure 2). Preclinical studies suggest that IL-17-mediated expression of cytokines and chemokines in structural cells contributes to neutrophilic inflammation, airway remodeling, loss of lung function and lung damage in COPD, CF, and asthma, and tumor proliferation and resistance to immunotherapy. Thus, therapeutic interventions addressing IL-17 cytokines and IL-17-signaling are a potential strategy to reduce pulmonary inflammation in chronic lung diseases, especially when combined with additional therapeutic approaches, such as immunotherapy in lung cancer. However, so far, clinical studies could not prove a general benefit of the blockade of IL-17 cytokines in chronic lung diseases. Thus, future research needs to identify patient cohorts that benefit from the therapeutic intervention in IL-17-signaling.

## Data Availability

Not applicable.
